# Clinical and laboratory features in patients with positive syphilis serology presenting with acute ischemic stroke or transient ischemic attack: a prospective cohort study

**DOI:** 10.1186/s12879-022-07700-z

**Published:** 2022-08-30

**Authors:** Pornpong Jitpratoom, Adhiratha Boonyasiri

**Affiliations:** 1 Division of Medicine, Chumphonkatudomsak Hospital, Chumporn, Thailand; 2 Department of Research and Development, Faculty of Medicine Siriraj Hospital, Mahidol University, Bangkok, Thailand

**Keywords:** Syphilis, Neurosyphilis, Latent syphilis, Meningovascular, Acute ischemic stroke, Transient ischemic attack, Alopecia, Generalized brain atrophy, Cerebrospinal fluid, Stroke outcome, *Treponema pallidum*

## Abstract

**Background:**

Neurosyphilis (NS) can lead to acute ischemic stroke (AIS) or transient ischemic attack (TIA). We compared the clinical characteristics and laboratory features among AIS and TIA patients who were syphilis-seronegative (control group) or had latent syphilis (LS) or NS to evaluate their stroke outcome.

**Methods:**

This prospective cohort study was conducted on patients who had recently suffered AIS or TIA. After serological syphilis screening, clinical and laboratory data were collected, and brain imaging and spinal tap (serologically syphilis-positive patients only) were performed. Stroke outcome was re-evaluated approximately three months later.

**Results:**

The 344 enrolled patients were divided into three groups: control group (83.7%), LS (13.1%), and NS (3.2%). A multivariate analysis revealed: 1) age of ≥ 70 years, generalized brain atrophy via imaging, and alopecia (adjusted odds ratio [AOR] = 2.635, 2.415, and 13.264, respectively) were significantly associated with LS vs controls; 2) age of ≥ 70 years (AOR = 14.633) was significantly associated with NS vs controls; and 3) the proportion of patients with dysarthria was significantly lower (AOR = 0.154) in the NS group than in the LS group. Regarding the NS patient cerebrospinal fluid (CSF) profile, only 2/11 cases had positive CSF-Venereal Disease Research Laboratory (VDRL) test results; the other nine cases were diagnosed from elevated white blood cell counts or protein levels combined with positive CSF fluorescent treponemal antibody absorption (FTA-ABS) test results. Regarding disability, the initial modified Rankin scale (mRS) score was lower in the control group than in the NS group (*p* = 0.022). At 3 months post-stroke, the mRS score had significantly decreased in the control (*p* < 0.001) and LS (*p* = 0.001) groups. Regarding activities of daily living, the 3-month Barthel Index (BI) score was significantly higher in control patients than in LS (*p* = 0.030) or NS (*p* = 0.002) patients. Additionally, the 3-month BI score was significantly increased in the control (*p* < 0.001) and LS (*p* = 0.001) groups.

**Conclusions:**

Because syphilis was detected in many AIS and TIA patients, especially those aged ≥ 70 years, routine serological syphilis screening may be warranted in this population. Patients with syphilitic infection had worse stroke outcomes compared with NS patients.

## Background

Syphilis is a sexually transmitted disease caused by infection with the spirochete *Treponema pallidum*. Syphilis incidence per 100,000 population in Thailand increased five times from 2.16 in year 2008 to 11.51 in year 2018 [1]. Also, between 2005 and 2011, a significant increase occurred in the annual prevalence of syphilis from 5.0% to 12.5%. Syphilis has been considered to be a major sexually transmitted disease in Thailand [2]. Infected individuals typically follow a disease course divided into primary, secondary, latent, and tertiary stages. Neurologic involvement, i.e., neurosyphilis (NS), occurs in up to 10% of patients with untreated syphilis [3]. NS is typically described as a late-stage manifestation, but neuroinvasion and neurological disease may occur at any stage of syphilis [4]. Meningovascular NS is an infectious inflammatory arteriopathy caused by the invasion of *T. pallidum* into the meninges, their arteries, and the surrounding arteries. Among NS patients, 14.09% develop ischemic stroke as a primary symptom, but only 0.31–0.25% of cases of acute ischemic stroke (AIS) and transient ischemic attack (TIA) are caused by NS [5–7].

At present, understanding of the prevalence and the clinical and laboratory characteristics of NS among patients with AIS or TIA is very limited; only a few studies on this topic have been conducted [6, 8]. To our knowledge, no studies have analyzed the outcome of such patients. Moreover, a gold standard test for detecting NS has not been established yet; in several studies [6–10], the diagnosis of NS was made on the basis of a cerebrospinal fluid (CSF) abnormality. Notably, AIS or TIA patients usually have risk factors for atherosclerosis. Therefore, in a patient who is serologically positive for syphilis, AIS or TIA might result from atherosclerosis unrelated to asymptomatic neurosyphilis, rather than from syphilitic meningovasculitis. In the present study, we aimed to estimate the prevalence and analyze the clinical and laboratory features and outcome of patients with positive syphilis serology (due to latent syphilis [LS] or meningovascular NS) in the population of patients presenting with AIS or TIA to determine the factor associated with the stroke outcome.

## Methods

This prospective cohort study was conducted at Chumphonkhetudomsakdi Hospital, Thailand, from November 2019 to November 2020. All patients over 15 years of age presenting within 7 days of newly onset AIS or TIA at our hospital during the study period were considered for study inclusion and screened for syphilis serology. Patients showing serological positivity for syphilis underwent a lumbar puncture. Patients were excluded from study participation if they showed signs or symptoms suggestive of increased intracranial pressure or had imaging results that indicated the same, or if they refused to undergo a lumbar puncture. Written informed consent was obtained from the patients or patients' surrogates. Baseline patient characteristics, including atherosclerotic risk factors, neurological status severity, laboratory diagnosis of stroke, computed tomography (CT) brain scans, and syphilis serological profile, were collected. The enrolled patients were classified into the following three groups: syphilis-seronegative (control group), LS, and NS.

A presumptive diagnosis of syphilis required the use of two laboratory serologic tests: a treponemal test and a nontreponemal test [11]. Serological testing for syphilis in this study was conducted via a chemiluminescence microimmunoassay (CMIA; for treponemal tests) and rapid plasma reagin (RPR; for non-treponemal tests) in all included patients. If the treponemal test is positive but the non-treponemal test is negative, the laboratory will perform a treponemal test different from the one used for initial testing [11]. We chose the fluorescent treponemal antibody absorption (FTA-ABS) test as an alternative treponemal test. NS was diagnosed in patients with positive syphilis serology, clinical features consistent with AIS or TIA, and a reactive cerebrospinal fluid-Venereal Disease Research Laboratory (CSF-VDRL) test result. In cases where a non-reactive CSF-VDRL test result occurred but the patient had an elevated CSF leukocyte count (> 5 white blood cells [WBCs]/mm^3^) or an elevated level of CSF protein (> 45 mg/dL), NS was still diagnosed if a CSF fluorescent treponemal antibody absorption (CSF FTA-ABS) test yielded a reactive result. In this study, LS was defined as having syphilis seropositivity in the absence of clinical manifestations of primary, secondary, or tertiary disease [11]. All included cases that did not fall into the LS or NS groups were categorized as controls.

For stroke outcome, modified Rankin scale (mRS) [12] and Barthel Index (BI) [13] scores were obtained at the time of enrollment (initial baseline scores). In the third month after the stroke, a telephone follow up was conducted. There is extensive evidence supporting the use of mRS and BI scores for stroke outcome assessment, and these scores have also been used to evaluate the validity and reliability of assessment administered over the telephone as compared with face-to-face assessment [14–20].

### Statistical analysis

All statistical analyses were performed using PASW Statistics version 18.0 (SPSS Inc., Chicago, IL, USA). Descriptive statistics were used to summarize demographic characteristics such as patient age and sex. Quantitative data are presented as the mean ± standard deviation or median (25th percentile, 75th percentile), and qualitative data are presented as the frequency (percentage). A chi-squared test or Fisher’s exact test was performed on categorical variables, such as patient sex, across the control group, LS, and NS groups. A one-way ANOVA or the Kruskal–Wallis H test was used to compare the quantitative data of the three groups. Unadjusted pairwise comparisons are reported, and once Bonferroni’s adjustment has been applied, the *p*-value required for significance is 0.016. All predictors identified as significant were subsequently tested in a multivariable analysis. Multinomial logistic regression was used to estimate the adjusted odds ratio (AOR) of the LS and NS groups with controls as the comparison group via a manual backward step. The CSF profiles of the LS and NS groups were compared using a Fisher’s exact test and Mann–Whitney U test. The Wilcoxon signed-rank test was used to compare the initial and 3-month mRS and BI scores.

### Ethics approval

The study was approved by the human research ethics committee of the Faculty of Medicine, Thammasat University (Ref: MTU-EC-OO-0-080/62) and informed consent obtained from each participant. All methods were carried out in accordance with relevant guidelines and regulations such as the Declaration of Helsinki, The Belmont Report, CIOMS Guidelines and the International Practice (ICH-GCP).

## Results

### Demographic characteristics of the AIS and TIA population

**Fig. 1:**
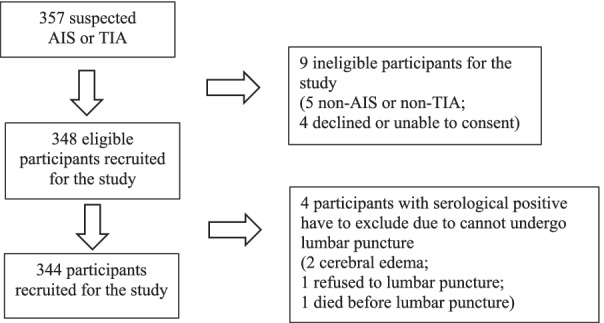
Flow diagram showing study recruitment

A total of 344 patients were recruited in this study (Fig. 1). The mean patient age was 65 (range: 17–97) years, and 60.8% of the participants were men. Most of the patients had an AIS (n = 327; 95.1%), and the rest had a TIA (n = 17, 4.9%). The most common atherosclerotic risk factor was hypertension (64.0%), followed by dyslipidemia (43.9%) and smoking (40.7%). The patients were categorized by the trial of ORG 10172 in acute stroke treatment (TOAST) classification [21] into five subtypes: (1) large-artery atherosclerosis (30.2%); (2) cardioembolism (16.3%); (3) small-vessel occlusion (46.5%); (4) stroke of other determined etiology (5.8%); and (5) stroke of undetermined etiology (1.2%).

### Comparison of the clinical characteristics and laboratory features of control, LS, and NS patients

Patients were divided into three groups according to their serological results: controls (83.7%), LS (13.1%), and NS (3.2%). For the HIV status, the testing was not routinely performed because it was not included in the study protocol, which raised concerns about patients' rights and appropriation in the AIS and TIA investigations. The results of a univariate analysis of the clinical characteristics and laboratory features of the three groups are shown in Table 1. A multivariate analysis revealed that, compared with controls, the factors significantly associated with LS were age of ≥ 70 years (AOR = 2.635), generalized brain atrophy on imaging (AOR = 2.415), and alopecia (AOR = 13.264) and the factor significantly associated with NS was age of ≥ 70 years (AOR = 14.633). The multivariate analysis also identified a significantly lower incidence of dysarthria among NS patients than among LS patients (AOR = 0.154).

**Table 1:** Clinical characteristics and laboratory features of patients without or with latent syphilis or neurosyphilis

	1. Controls	2. Latent syphilis	3. Neurosyphilis	*p*-value^#^	P1 (1 vs 2)	P2 (1 vs 3)	P3 (2 vs 3)
n	288	45	11				
Male sex, n (%)	171 (59.4%)	30 (66.7%)	8 (72.7%)	0.460	0.35	0.38	0.70
Age, mean ± SD (years)	63.07 ± 15.20	73.09 ± 12.55	79.82 ± 8.54	< 0.001	< 0.001	< 0.001	0.099
TIA, n (%)	17 (5.9%)	0 (0.0%)	0 (0.0%)	0.262	0.14	1.00	
HIV-positive, n/tested patients (%)	1/66 (1.5%)	0/25 (0.0%)	0/5 (0.0%)	1.000	1.00	1.00	
*Stroke risk factor*							
Diabetes mellitus, n (%)	90 (31.3%)	9 (20.0%)	2 (18.2%)	0.217	0.12	0.36	0.89
Hypertension, n (%)	184 (63.9%)	28 (62.2%)	8 (72.7%)	0.808	0.83	0.55	0.51
Chronic kidney disease, n (%)	53 (18.4%)	14 (31.1%)	4 (36.4%)	0.062	0.048	0.14	0.74
Dyslipidemia, n (%)	128 (44.4%)	18 (40.0%)	5 (45.5%)	0.851	0.58	0.95	0.74
Atrial fibrillation, n (%)	47 (16.3%)	6 (13.3%)	3 (27.3%)	0.532	0.61	0.34	0.26
Smoking, n (%)	121 (42.0%)	16 (35.6%)	3 (27.3%)	0.481	0.52	0.37	0.73
*Stroke symptoms*							
Motor weakness, n (%)	263 (91.3%)	42 (93.3%)	10 (90.9%)	1.000	1.00	1.00	1.00
Visual disturbance, n (%)	70 (24.3%)	8 (17.8%)	3 (27.3%)	0.604	0.34	0.82	0.48
Vertigo, n (%)	86 (29.9%)	8 (17.8%)	2 (18.2%)	0.187	0.094	0.40	0.97
Sensory abnormalities, n (%)	140 (48.6%)	18 (40.0%)	7 (63.6%)	0.321	0.28	0.33	0.16
Language, n (%)	61 (21.2%)	13 (28.9%)	1 (9.1%)	0.296	0.30	0.47	0.26
Dysarthria, n (%)	212 (73.6%)	35 (77.8%)	5 (45.5%)	0.089	0.55	0.040	0.033
*Location of acute lesion from imaging*				0.591	0.880	0.27	0.28
Middle cerebral artery, n (%)	97 (33.7%)	15 (33.3)	2 (18.2%)				
Anterior cerebral artery, n (%)	4 (1.4%)	0 (0.0%)	0 (0.0%)				
Posterior cerebral artery, n (%)	10 (3.5%)	0 (0.0%)	0 (0.0%)				
Basilar artery, n (%)	8 (2.8%)	1 (2.2%)	1 (9.1%)				
Subcortical white matter, n (%)	41 (14.2%)	9 (20.0%)	3 (27.3%)				
Posterior limb of internal capsule, n (%)	28 (9.7%)	6 (13.3%)	0 (0.0%)				
Basal ganglia, n (%)	33 (11.5%)	4 (8.9%)	0 (0.0%)				
Others, n (%)	43 (14.9%)	8 (17.8%)	3 (27.3%)				
Not identified, n (%)	24 (8.3%)	2 (4.4%)	2 (18.2%)				
Old cerebral infarction on imaging, n (%)	103 (35.8%)	26 (57.8%)	5 (45.5%)	0.017	0.005	0.51	0.46
Generalized brain atrophy on imaging, n (%)	100 (34.7%)	28 (62.2%)	8 (72.7%)	< 0.001	< 0.001	0.010	0.51
*History of syphilis symptoms*							
Chancre, n (%)	9 (3.1%)	3 (6.7%)	0 (0.0%)	0.470	0.21	1.00	1.00
Oral ulcer, n (%)	8 (2.8%)	1 (2.2%)	0 (0.0%)	1.000	1.00	1.00	1.00
Rash, n (%)	16 (5.6%)	4 (8.9%)	0 (0.0%)	0.472	0.33	1.00	0.58
Alopecia, n (%)	7 (2.4%)	8 (17.8%)	0 (0.0%)	< 0.001	< 0.001	1.00	0.33
Lymphadenopathy, n (%)	0 (0.0%)	1 (2.2%)	0 (0.0%)	0.163	0.14		1.00
Urogenital wart, n (%)	6 (2.1%)	1 (2.2%)	0 (0.0%)	1.000	1.00	1.00	1.00
Mucocutaneous lesion, n (%)	3 (1.0%)	1 (2.2%)	0 (0.0%)	0.510	0.44	1.00	1.00
At least one previous syphilitic symptom, n (%) *	43 (14.9%)	13 (28.9%)	0 (0.0%)	0.021	0.020	0.17	0.042
*Initial evaluation*							
Initial plasma glucose (mg/dL), median (P25, P75)	106 (90, 143)	107 (95, 145)	100 (91, 127)	0.486	0.29	0.64	0.28
Hemoglobin (g/dL), median (P25, P75)	13.00 (12.00, 14.20)	13.00 (12.00, 14.00)	12.80 (10.80, 14.00)	0.478	0.28	0.52	0.99
Hematocrit (%), median (P25, P75)	39.00 (35.00, 42.45)	37.70 (32.00, 39.80)	38.00 (32.40, 42.00)	0.061	0.019	0.61	0.46
White blood cell count (cells/mm^3^), median (P25, P75)	8445 (6880, 10,905)	8680 (6490, 11,260)	10,060 (6640, 11,140)	0.913	0.85	0.70	0.74
Platelet count (cells/mm^3^), median (P25, P75)	252,000 (210,000, 304,750)	229,000 (190,500, 306,000)	205,000 (165,000, 252,000)	0.027	0.092	0.026	0.27
PT (s), median (P25, P75)	10.95 (10.00, 11.50)	11.00 (10.30, 11.60)	11.00 (10.80, 11.60)	0.470	0.28	0.50	0.98
PTT (s), median (P25, P75)	24.00 (22.00, 26.00)	23.50 (22.80, 25.30)	22.80 (21.00, 25.50)	0.418	0.65	0.22	0.19
LDL (mg/dL), median (P25, P75)	111.0 (80.5, 141.0)	104.0 (75.0, 126.0)	87.0 (84.0, 109.0)	0.355	0.58	0.18	0.27
BUN (mg/dL), median (P25, P75)	14 (11, 17)	15 (11, 18)	18 (13, 26)	0.072	0.16	0.058	0.26
Cr (mg/dL), median (P25, P75)	0.89 (0.70, 1.00)	0.93 (0.80, 1.14)	0.90 (0.84, 1.54)	0.025	0.030	0.073	0.58
GFR (ml/min), median (P25, P75)	87 (66, 98)	74 (56, 88)	78 (35, 84)	< 0.001	0.003	0.011	0.35

*BUN* blood urea nitrogen, *Cr* creatinine, *GFR* glomerular filtration rate, *HIV* human immunodeficiency virus, *INR* international normalized ratio, *LDL* low-density lipoprotein, *n* number, *P1* controls compared with latent syphilis, *P2* controls compared with neurosyphilis, *P3* latent syphilis compared with neurosyphilis, *P25 *25th percentile, *P75* 75th percentile, *PT* prothrombin time, *PTT* partial thromboplastin time, *SD* standard deviation, *TIA* transient ischemic attack. Unadjusted pairwise comparisons are reported, and once Bonferroni’s adjustment has been applied, the p-value required for denoting statistical significance is 0.016

*Symptoms include chancre, oral ulcer, rash, alopecia, lymphadenopathy, urogenital wart, and mucocutaneous lesion.

^#^A Chi-squared test or Fisher’s exact test was performed to assess the association between qualitative clinical characteristics and laboratory features of the patients, such as patient sex, and three syphilis groups according to their serological results: control, LS, and NS. A one-way ANOVA or the Kruskal–Wallis H test was used to compare mean or median of the quantitative data between the three groups

### Clinical characteristics, CSF profiles, and therapeutic treatment of syphilis patients

The clinical characteristics and CSF profiles of the 11 NS patients are shown in Table 2. The CSF profiles of the NS patients revealed that the mean WBC count was 4.9 cells/mm^3^, the lymphocyte percentage was 72%, the level of CSF protein was 111 mg/dL, and the CSF/serum glucose ratio was 0.57. The CSF-VDRL tests results were reactive in only two NS cases (the other nine NS cases were diagnosed on the basis of an elevated WBC count or protein level in combination with a reactive CSF FTA-ABS test result). A comparison of the CSF profiles of the LS and NS patients (Table 3) revealed no significant differences in the WBC count, but the CSF protein level in the NS group was significantly higher than that in the LS group (*p* = 0.013). All NS patients were treated with 24 million units of aqueous crystalline penicillin G per day, administered intravenously as 4 million units every 4 h for 14 days. Among the 45 LS patients, 36 patients were injected with benzathine penicillin G intramuscularly as three doses of 2.4 million units at 1-week intervals, 6 patients took 100 mg of doxycycline orally twice daily for 28 days, 2 cases were fatal before treatment was initiated, and the remaining case refused treatment.

**Table 2:** Clinical characteristics and CSF profiles of neurosyphilis patients (all CMIA-serological positive)

Case	Age (years)	Sex	Symptoms	Underlying conditions	Serology	WBC (cells/mm^3^)	N%	L%	RBC (cells/mm^3^)	Protein (mg/dL)	Sugar (mg/dL)	CSF/serum glucose ratio	CSF VDRL	CSF FTA-ABS	Location of lesion	3-month outcome
1	85	M	hemiparesis, hemianesthesia	DM, HT, smoking	RPR: non-reactive FTA-ABS: reactive	1	0	100	0	83	66	0.46	NR	R	no definite lesion	mRS: 4, BI: 40
2	85	F	hemiparesis	HT, DLP, CAD	RPR titer 1:4	1	0	100	0	46	68	0.58	NR	R	no definite lesion	mRS: 6, BI: 0
3	77	M	hemiparesis, blurred vision, vertigo, hemianesthesia	HT	RPR titer 1:4	7	29	71	4,000	71	72	0.55	1:4	NR	brainstem	mRS: 4, BI: 90
4	63	M	hemiparesis, hemianesthesia	HT, DLP, AF	RPR: weakly reactive	4	50	50	1,000	139	78	0.62	NR	R	BA territory	mRS: 5, BI: 0
5	80	M	hemiparesis, blurred vision, vertigo, hemianesthesia	DM, HT	RPR: weakly reactive	1	100	0	0	170	63	0.52	NR	WR	brainstem	mRS: 2, BI: 100
6	94	F	aphasia	HT, DLP	RPR: non-reactive FTA-ABS: reactive	2	50	50	0	390	80	0.61	NR	R	subcortical white matter	mRS: 6, BI: 0
7	78	M	hemiparesis, dysarthria, hemianesthesia	Alcoholism	RPR: non-reactive FTA-ABS: reactive	17	7	93	1,000	78	58	0.68	NR	R	thalamus	mRS: 4, BI: 65
8	74	M	hemiparesis, dysarthria	DLP, smoking, CVD	RPR titer 1:16	18	67	33	18,000	51	74	0.61	NR	WR	MCA territory	mRS: 5, BI: 0
9	72	M	hemiparesis, dysarthria, hemianesthesia	smoking	RPR titer 1:1	1	0	100	0	90	69	0.62	NR	R	subcortical white matter	mRS: 2, BI: 95
10	89	M	hemiparesis, blurred vision	HT, DLP, CAD	RPR titer 1:1	1	0	100	0	59	64	0.41	NR	R	subcortical white matter	mRS: 4, BI: 40
11	81	F	hemiparesis, dysarthria, hemianesthesia	HT, AF	RPR: non-reactive FTA-ABS: reactive	1	0	100	0	50	65	0.68	1:2	NR	MCA territory	mRS: 5, BI: 0
Mean ± SD						4.91 ± 6.5	27 ± 34	72 ± 34	2181 ± 5381	111 ± 100	68 ± 6.6	0.57 ± 0.085				

*AF* atrial fibrillation, *BA* basilar artery, *BI* barthel index, *CAD* coronary artery disease, *CMIA* chemiluminescent microparticle immunoassay, *CSF* cerebrospinal fluid, *CVD* cerebrovascular disease, *DLP* dyslipidemia, *DM* diabetes mellitus, *F* female, *FTA-ABS* fluorescent treponemal antibody absorption, *HT* hypertension, *L* lymphocyte, *M* male, *MCA* middle cerebral artery, *mRS* modified Rankin scale, *N* neutrophil, *NR* non-reactive, *R* reactive, *RBC* red blood cell, *RPR* rapid plasma reagin, *SD* standard deviation, *VDRL* Venereal Disease Research Laboratory test, *WBC* white blood cell, *WR* weakly reactive

**Table 3:** Comparison of the CSF profiles of latent syphilis and neurosyphilis patients

	Total	Latent syphilis	Neurosyphilis	*p*-value^#^
	n = 56	n = 45	n = 11	
WBC (cells/mm^3^), median (P25, P75)	1 (1, 4)	1 (1, 3)	1 (1, 7)	0.471
N (%), median (P25, P75)	0 (0, 50)	0 (0, 50)	7 (0, 50)	0.714
L (%), median (P25, P75)	67 (28, 100)	67 (0, 100)	93 (50, 100)	0.238
RBC (cells/mm^3^), median (P25, P75)	0 (0, 1000)	0 (0, 1000)	0 (0, 1000)	0.713
Protein (mg/dL), median (P25, P75)	57 (42, 80)	54 (40, 71)	78 (51, 139)	0.013
Sugar (mg/dL), median (P25, P75)	66 (59, 79)	65 (57, 79)	68 (64, 74)	0.657
CSF/serum glucose ratio, median (P25, P75)	1 (1, 1)	1 (1, 1)	1 (1, 1)	0.665
CSF-VDRL				0.036
Non-reactive, n (%)	54 (96.4%)	45 (100.0%)	9 (81.8%)	
1:2, n (%)	1 (1.8%)	0 (0.0%)	1 (9.1%)	
1:4, n (%)	1 (1.8%)	0 (0.0%)	1 (9.1%)	
CSF FTA-ABS				< 0.001
Negative, n (%)	45 (80.4%)	43 (95.6%)	2 (18.2%)	
Reactive, n (%)	7 (12.5%)	0 (0.0%)	7 (63.6%)	
Weakly reactive, n (%)	4 (7.1%)	2 (4.4%)	2 (18.2%)	

*CSF* cerebrospinal fluid, *FTA-ABS* fluorescent treponemal antibody absorption, *L* lymphocyte; P25, 25th percentile, *N* neutrophil, *n* number, *P75* 75th percentile, *RBC *red blood cell, *VDRL* Venereal Disease Research Laboratory test, *WBC* white blood cell

^#^Fisher’s exact test was performed to assess the association between qualitative CSF profile and two syphilis groups: Latent syphilis and Neurosyphilis. Mann–Whitney U test was used to compare the median of the quantitative CSF profiles between the two groups

### Comparison of disability and activities of daily living (ADL) outcomes among the control, LS, and NS groups

After study enrollment, the initial mRS and BI scores were first recorded, and then the investigation for syphilitic infection was performed, followed by treatment if necessary. An evaluation was conducted via telephone three months later. The mRS scores, which represent the degree of disability due to stroke and range from 0 (no symptoms) to 6 (death), at the initial and 3-month timepoints are compared in Table 4. The initial mRS score in the control group was significantly lower than that in the NS group (*p* = 0.022), reflecting a mild disability at the initial evaluation of patients without syphilis. The 3-month mRS score was significantly decreased from the baseline score in the control (*p* < 0.001) and LS (*p* = 0.001) groups, indicating good recovery from disability in these patients. Additionally, the BI score was used to measure performance in ADL, with scores ranging from 0 (totally dependent) to 100 (normal ADL). There was no significant difference in the initial BI scores among the three groups. However, the 3-month post-stroke BI score was significantly higher in the control group than in the LS (*p* = 0.003) and NS (*p* = 0.002) groups, reflecting better ADL performance at this timepoint among patients without syphilis. The 3-month post-stroke median BI score was significantly increased from the initial BI score in the control (*p* < 0.001) and LS (*p* = 0.001) groups, indicating good recovery with respect to ADL performance among these patients.

**Table 4:** Outcome comparison of patients without and with latent syphilis and neurosyphilis

	Controls	Latent syphilis	Neurosyphilis	*p*-value^#^	P1	P2	P3
Modified Rankin Scale*							
Initial (points), median (P25, P75)	4 (3, 5)	5 (4, 5)	5 (4, 5)	0.022	0.095	0.022	0.12
3 months (points), median (P25, P75)	2 (1, 4)	3 (2, 5)	4 (4, 5)				
* p*-value (difference between initial and 3-month scores)	< 0.001	0.001	0.236				
Change from initial (3-month score–initial score), median (P25, P75)	− 1 (− 2, 0)	− 1 (− 2, 0.5)	0 (− 1, 0)	0.077	0.124	0.076	0.448
Barthel Index**							
Initial (points), median (P25, P75)	50 (30, 75)	50 (20, 65)	55 (10, 60)	0.204	0.14	0.27	0.69
3 months (points), median (P25, P75)	100 (50, 100)	90 (5, 100)	40 (0, 90)	0.001	0.030	0.002	0.095
* p*-value (difference between initial and 3-month scores)	< 0.001	0.001	0.838				
Change from initial (3-month score–initial score), median (P25, P75)	25 (0, 44)	25 (− 5, 40)	− 10 (− 20, 25)	0.028	0.274	0.012	0.076

*P1* controls compared with latent syphilis, *P2* controls compared with neurosyphilis, *P3* latent syphilis compared with neurosyphilis, *P25* 25th percentile, *P75* 75th percentile

^#^The Kruskal–Wallis H test was used to compare median of the outcomes between the three groups

*Scores on the modified Rankin scale (mRS) range from 0 (no symptoms) to 6 (death)

**The Barthel Index (BI) scores range from 0 (totally dependent) to 100 (normal activities of daily living)

## Discussion

Meningovascular NS is a rare cause of stroke that occurs in approximately 10% of NS cases and in approximately 3% of all syphilis cases (22, 23). Previous studies conducted in Australia and Portugal, countries with a very low prevalence of syphilis, found the prevalence of positive syphilis serology results among patients who presented with an acute stroke to be 4% and 3.72%, respectively (6, 24, 25). In Thailand, a 2011 study found that the prevalence of syphilis among patients who presented with an acute stroke was 8.4% (11), which is only half of the syphilis prevalence found by our present study (16.3%). Our data indicate that syphilis remains a major problem in Thailand.

Meningovascular NS often presents with a stroke among patients younger than 50 years of age and typically occurs within 10 years of syphilitic infection (6, 26–28). Regarding studies conducted before the year 2000 (29, 30), up to 74% of meningovascular NS patients who presented with a stroke were under the age of 50 years. However, the epidemiology of NS has changed over the last decade. A recent study conducted in Portugal revealed a mean age of 76 years in NS patients who presented with a stroke and reported several cerebrovascular risk factors for ischemic stroke. This finding showed a trend similar to that of our study, in which the median age of meningovascular NS patients was 80 years old. Moreover, the multivariate analysis comparing patients without and with LS or NS (Table 3) revealed that an age of ≥ 70 years was an independent factor associated with LS and NS. This result emphasizes that in the AIS and TIA population, the age of most syphilis-infected patients was higher than that of the typical patient with meningovascular NS, as described above. Therefore, routine serological screening for syphilis may be warranted in AIS and TIA patients aged 70 years or older, especially in regions with a high prevalence of syphilis.

Although the incidence of meningovascular NS continues to rise, only a few studies have focused on syphilis in elderly patients with typical ischemic stroke or TIA who present with atherosclerosis and cardiovascular risk factors. Through neuroimaging, we found that generalized brain atrophy was a factor associated with LS and showed a trend towards being a factor associated with NS (in the univariate analysis, the comparison of control group with NS yielded a *p*-value of 0.01, but this difference was not significant in the multivariate analysis). Several case reports have shown generalized brain atrophy via imaging in NS patients who presented with a progressive cognitive decline (24, 31–33). However, for LS, there was no obvious evidence for an association with generalized brain atrophy. Therefore, this result requires confirmation from additional studies.

Alopecia has historically been associated with syphilis infection (34–36). In the present study, alopecia was a factor significantly associated with LS as compared with controls. However, no patient in the NS group presented with this symptom, which may be a consequence of the small sample size in that group.

The multivariate analysis comparing LS and NS revealed that the proportion of patients with dysarthric symptoms of stroke was significantly lower in the NS group than in the LS group (AOR = 0.154). Previous studies have reported that dysarthria is a symptom of NS, (24, 33, 37), but the observations from the present work do not support that finding; however, this discrepancy may stem from the confounding effect of our small sample size.

To further investigate abnormalities of the CSF profiles in our study, we allowed the diagnosis of NS with TIA or AIS from criteria that was proposed by the previous study in Thailand (7). There is a frequency of absence of CSF-cell elevation in the majority of our NS cases. The cause is not yet clear. But it may be assumed that one of the consequences of ageing is a decline in the immune response (mean age of NS was about 80 years old) (38–40). In 4 NS patients who had elevated red blood cells (RBC) in their CSF from traumatic tap, only one of them had CSF VDRL positivity with CSF RBCs of 4,000 cells/mm^3^, which is rarely a false positive result because, from Izzat et al. data, the amounts of CSF RBCs required to cause the CSF conversion have to be at least 12,000 cells/mm^3^ (41). The other 3 RBC contaminated NS patients had CSF VDRL negative with CSF FTA-ABS positive. When blood is contaminated, a CSF FTA-ABS is frequently false-positive, but in the context of a consistent clinical scenario, diagnosis and treatment for NS should be given (11, 42–45). Only 18.2% of our patients (n = 2/11) with TIA or AIS related to NS had positive CSF-VDRL results; thus, combining CSF VDRL results with CSF FTA-ABS results was useful to diagnose NS in AIS and TIA patients.

The degree of stroke-related disability in the NS group was initially severe compared with that in the control group, and the severity of disability did not significantly improve over the subsequent three months. Moreover, the performance of ADL at 3 months post-stroke in the NS group was significantly worse than that in the control group and had improved significantly less from the baseline assessment compared with the control and LS groups. In LS patients, the ADL performance at 3-months post-stroke was significantly worse than that in control patients. This stroke outcome demonstrates that an occult syphilitic infection is associated with a poor recovery, which may be from systemic infection combined with ageing and cerebral infarction pushing individual microglia toward proinflammatory phenotypes, compromising the connectivity of neuronal networks (46, 47). This effect of persistent immune activation in the brain can result in neurodegeneration and a decreased ability to improve (48).

There are a few limitations to our study. The number of NS patients was small and may not have been sufficient to demonstrate many factors associated with having NS. However, we were able to identify some associated factors, such as old age, alopecia, and generalized brain atrophy, that could be used to trigger clinical suspicion of NS among patients with AIS or TIA. A multicenter prospective study and long-term follow-up are needed to provide further support for these associations.

## Conclusions

This study displayed interesting hint characteristics which indicate to explore history and serological screening for syphilis in the acute ischemic cerebrovascular population. The AIS and TIA patients with one or more of aging more than seventy years old, a history of alopecia and generalized brain atrophy on the imaging were at risk for the *T. pallidum* infection. During the investigation for diagnosis of NS which based on CSF analysis, sending CSF VDRL and CSF FTA-ABS test at the same time was useful for confirming an NS diagnosis in a real-world situation which can avoid suffering from repeat lumbar puncture due to the low rate of CSF VDRL test result-positivity. Lastly, both NS and LS patients have a worse stroke consequence as compared with control patients that demonstrate by the mRS and BI score. From the electronic literature review, this is the first study that has shown the impact of the syphilitic infection on the prognosis of the AIS and TIA patients, which is the new evidence of connectivity between the infectious disease and the cerebrovascular disease.

## Data Availability

Individual level data cannot be shared publicly because of patient confidentiality under current Thai legislation. The data that support the findings of this study are available from Chumphonkatudomsak Hospital, but restrictions apply to the availability of these data, which were used under license for the current study, and so are not publicly available. Data are however available from the authors upon reasonable request and with permission of Chumphonkatudomsak Hospital and the appropriate ethics committee.

## Abbreviations

AIS: Acute ischemic stroke
AF: Atrial fibrillation
ANOVA: Analysis of variance
AOR: Adjusted odds ratio
BA: Basilar artery
BI: Barthel index
BUN: Blood urea nitrogen
CAD: Coronary artery disease
CIOMS: Council for International Organizations of Medical Sciences
CMIA: Chemiluminescence microimmunoassay
Cr: Creatinine
CSF: Cerebrospinal fluid
CSF FTA-ABS: Cerebrospinal fluid fluorescent treponemal antibody absorption
CSF-VDRL: Cerebrospinal Fluid-Venereal Disease Research Laboratory
CT: Computed tomography
CVD: Cerebrovascular disease
DLP: Dyslipidemia
DM: Diabetes mellitus
F: Female
FTA-ABS: Fluorescent treponemal antibody absorption
GCP: Good clinical practice
GFR: Glomerular filtration rate
HIV: Human immunodeficiency virus
HT: Hypertension
ICH: International Conference on Harmonization
INR: International normalized ratio
IL: Illinois
L: Lymphocyte
LDL: Low-density lipoprotein
LS: Latent syphilis
M: Male
MCA: Middle cerebral artery
mRS: Modified Rankin Scale
n: Number
N: Neutrophil
NR: Non-reactive
NS: Neurosyphilis
PASW: Predictive analytics software
PT: Prothrombin time
PTT: Partial thromboplastin time
R: Reactive
RBC: Red blood cell
RPR: Rapid plasma reagin
SD: Standard deviation
SPSS: Statistical Package for the Social Sciences
TIA: Transient ischemic attack
TOAST: Trial of ORG 10172 in acute stroke treatment
*T. pallidum*: *Treponema pallidum*
USA: United States of America
VDRL: Venereal Disease Research Laboratory
WBCs: White blood cells
WR: Weakly reactive
